# New intervention strategy for postoperative fatigue syndrome in elderly patients with colorectal cancer: a clinical hypothesis study based on vagus nerve stimulation

**DOI:** 10.3389/fmed.2025.1588850

**Published:** 2025-06-02

**Authors:** Xuefeng Yin, Shuang Qiao, Ling Zhang, Zhonghua Li, Qin Zhang, Yu Shen, Keyu Fan, Mingxia Liu, Dongxu Wang, Ya Cao, Yuxuan Zhang, Lu Qian, Danru Wu, Jingqiu Wei, Ying Yang, He Liu

**Affiliations:** ^1^Department of Anesthesiology and Clinical Research Center for Anesthesia and Perioperative Medicine and Key Laboratory of Anesthesia and Analgesia Application Technology, Huzhou Central Hospital, The Fifth School of Clinical Medicine of Zhejiang Chinese Medical University, Huzhou, Zhejiang, China; ^2^Huzhou Central Hospital, The Affiliated Central Hospital of Huzhou University, Huzhou, Zhejiang, China; ^3^Affliated Huzhou Hospital, Zhejiang University School of Medicine, Huzhou, Zhejiang, China; ^4^Department of Education and Training, Huzhou Central Hospital, The Fifth School of Clinical Medicine of Zhejiang Chinese Medical University, Huzhou, Zhejiang, China; ^5^Department of Anesthesiology, The Affiliated Hospital of Xuzhou Medical University, Xuzhou, Jiangsu, China; ^6^HuzhouKey Laboratory of Basic Research and Clinical Translation for Neuromodulation, Huzhou Central Hospital, The Fifth School of Clinical Medicine of Zhejiang Chinese Medical University, Huzhou, Zhejiang, China; ^7^Ministry of Education Key Laboratory for Neuroinformation, University of Electronic Science and Technology of China, Chengdu, Sichuan, China

**Keywords:** transcutaneous auricular vagus nerve stimulation, postoperative fatigue syndrome, cholinergic anti-inflammatory pathway, postoperative recovery, colorectal cancer

## Abstract

Postoperative fatigue syndrome (POFS) comprises symptoms including fatigue, insomnia, inattention, depression, tension, and anxiety following surgery. These manifestations encompass exhaustion, weakness, malaise, and emotional disturbances, impacting hospital stay duration, quality of life, rehabilitation progress, and work performance. While the etiology of POFS remains complex, recent evidence suggests that external stimuli may induce pro-inflammatory cytokine release, leading to fatigue. Surgical procedures trigger an inflammatory reaction that stimulates the nervous system, generating fatigue symptoms. Both animal and human studies demonstrate that vagus nerve stimulation (VNS) can reduce pro-inflammatory cytokine production by activating the cholinergic anti-inflammatory pathway (CAP). Considering the pivotal role of inflammation in the development of POFS and the vagus nerve's capacity to modulate inflammatory responses, we hypothesize that transcutaneous auricular vagus nerve stimulation (taVNS) holds significant potential for alleviating POFS in elderly patients undergoing colorectal cancer surgery. In this paper, we propose a hypothetical scheme to validate this hypothesis through the application of taVNS in future clinical studies.

## 1 Introduction

Enhanced Recovery After Surgery (ERAS) is a patient-centered approach to surgical care that has emerged as a transformative paradigm in perioperative management, aiming to optimize patient outcomes, expedite recovery, and minimize hospital stays (1). After surgery, individuals commonly experience POFS, which can be affected by factors such as the type of surgery, the healing process, and emotional strain. POFS is a normal bodily response to the stress of surgery, regardless of how extensive or complicated the procedure was, and it presents as weakness, tiredness, lethargy, and a decreased ability to engage in daily activities. The duration of post-surgical fatigue can vary widely, impacting daily life and healing. Primary clinical manifestations of POFS include fatigue, sleep disturbances, impaired attention, reduced activity levels, and emotional abnormalities (2). In major abdominal surgery, is reported to be as high as 92%, and it persists in 10% of patients even after 3 months (3), Due to the decline of physical function, the incidence of POFS is higher in elderly patients, posing significant challenges for clinicians in addressing this complication within surgical populations because of the lack of clarity regarding its etiology and mechanism, along with limited treatment options available for symptom relief.

Although the mechanism of POFS is not clear, the impact of surgery as an invasive operation has attracted more and more attention because exogenous stimulation has confirmed that it can produce related pro-inflammatory cytokines. Surgical trauma serves as an exogenous stimulus that elicits an inflammatory response. The subsequent release of inflammatory mediators further activates the nervous system, thereby contributing to the manifestation of fatigue symptoms (4). Growing evidence has demonstrated the close association between cytokines and the occurrence and progression of POFS. A meta-analysis (5) comparing open and laparoscopic colorectal cancer surgery revealed a linear correlation between inflammatory factors, such as interleukin-6(IL-6), IL-1β, tumor necrosis factor-α (TNF-α) levels, neopterin immune index, and POFS. An increasing number of studies have emphasized the important roles that inflammatory factors and their associated immune mechanisms play in the onset of POFS. A systematic retrospective analysis (6) on muscle performance recovery in elderly patients after surgery indicated that a higher postoperative inflammatory response is significantly linked to poorer muscle strength and preoperative fatigue. Preoperative administration of steroids or glucocorticoids for inflammation control can effectively relieve postoperative fatigue in elderly patients. Hence, surgical-induced inflammation may be a key factor in the pathophysiology of POFS.

The nervous system has the ability to regulate inflammatory responses by suppressing the release of TNF-α from macrophages through vagus nerve stimulation. This anti-inflammatory effect is mediated by the interaction between acetylcholine (ACh) and nicotinic acetylcholine receptor (α7nAChR), which contains the α7 subunit on macrophages, constituting what is known as the CAP (7). With an increasing recognition of CAP, there is a growing interest in comprehensive inflammation control that extends beyond solely targeting cytokines. ACh serves as a vital neurotransmitter for the vagus nerve and has been demonstrated to significantly inhibit pro-inflammatory cytokine release, including TNF, IL-1β, IL-6, and IL-18, however, it does not inhibit anti-inflammatory cytokines like IL-10 (8). This highlights the crucial role played by CAP.

## 2 The hypothesis

We hypothesize that taVNS may alleviate postoperative fatigue in elderly patients undergoing colorectal cancer surgery by modulating the inflammatory response. To date, this hypothesis has not been fully articulated nor validated through clinical trials. Our rationale for proposing this hypothesis is based on the recognized role of inflammation in the pathogenesis of POFS and the vagus nerve's mediation of the inflammatory response via the CAP.

## 3 Rationale for the theory

### 3.1 Central mechanisms of POFS

Currently, the mechanism of POFS, particularly the central fatigue mechanism, remains incompletely understood. Central fatigue primarily manifests in mental and psychological states. Recent animal studies have indicated that this mechanism involves the central metabolic pathway of 5-hydroxytryptamine (5-HT), the N-methyl-D-aspartate (NMDA) receptor pathway, and the inflammatory signaling pathway. The neurotransmitter 5-HT is essential in the central nervous system for the regulation of pain, sleep, body temperature, and emotions. Increased levels of 5-HT are thought to be a contributing factor to central fatigue. Tryptophan, a crucial amino acid predominantly found in the central nervous system, is closely involved in metabolism as it acts as a precursor for the synthesis of 5-HT. Surgical trauma-induced stress response exacerbates systemic catabolism, thereby increasing the concentration of free tryptophan in peripheral blood. This elevation facilitates the transport of tryptophan across the blood-brain barrier, leading to an accumulation of tryptophan in the brain. Consequently, this enhances metabolic activity and promotes the conversion of tryptophan into 5-HT (9, 10). In addition to stimulating 5-HT synthesis, elevated tryptophan levels may also activate NMDA receptors, potentially inducing neurotoxic effects that result in neuronal damage, dysfunction, and postoperative fatigue syndrome (11). Inflammatory cytokines are released into circulation following surgical injury and transmitted to the central nervous system through blood flow where they activate tryptophan metabolism via NMDA receptor pathways contributing to postoperative fatigue as well (12). Liu et al.'s (4) study reported that early postsurgical trauma activates classic inflammatory signaling pathways such as p38 MAPK and NF-κB within the rat hippocampus triggering a central inflammatory response associated with central fatigue.

### 3.2 Inflammatory effects of the vagus nerve

Both the afferent and efferent vagus nerves have been demonstrated to be involved in the regulation of inflammatory responses. VNS enhances ACh release, which subsequently binds to a7nAChR on macrophages, leading to the activation of CAP. This effect directly inhibits the production and release of pro-inflammatory cytokines (13). In lipopolysaccharide (LPS)-stimulated human macrophage cultures, ACh has been shown to suppress TNF synthesis through a posttranscriptional mechanism and inhibit the secretion of IL-1β, IL-6, and IL-8 without interfering with the release of the anti-inflammatory cytokine IL-10 (14).

### 3.3 CAP and VNS in inflammatory disease

So far, a considerable number of studies have reported the role of CAP-related interventions in inflammatory response-related pathological conditions, including sepsis, organ ischemia-reperfusion, pancreatitis, acute lung injury (ALI) and other inflammatory responses (15–18). In experimental sepsis, VNS is more effective in eliminating harmful pathogens, reducing inflammatory response and organ damage, and improving the survival rate of polymicrobial septic peritonitis models (19). Additionally, Borovikova et al. (20) reported the application of VNS in sepsis in 2,000 and found that VNS could alleviate the systemic inflammatory response in LPS-induced septic rats. A recent clinical study reported that patients with sepsis, after taVNS treatment for 5 days, can significantly reduce the levels of pro-inflammatory cytokines TNF-α and IL-1β in the circulation and can improve the levels of anti-inflammatory cytokines IL-10 (21). Furthermore, CAP has exhibited protective effects against ischemia/reperfusion injury in the kidney, liver, lung, and intestine by suppressing excessive inflammatory responses (17). In an experimental pancreatitis model, Huang et al. found that dexmedetomidine reduced systemic inflammatory response and local pancreatic injury caused by pancreatitis in rats through CAP (16). Li et al. found that CAP helps to reduce the inflammatory response in ALI by regulating the maturation, phenotype, and number of dendritic cells (DCs), conventional DCs, and conventional DCs2 (type 2 conventional DCs) (22). Previous studies have also shown that cholinergic agonists not only downregulate TNF-mediated inflammatory responses and lower plasma TNF-α levels but also increase plasma levels of anti-inflammatory substances such as IL-10 and glucocorticoids (23, 24).

### 3.4 VNS and inflammatory bowel disease (IBD)

Many studies have reported that VNS exhibits anti-inflammatory effects in animal models of colitis and in clinical experimental studies of human inflammatory bowel disease. In terms of animal models, Zhao et al. (25) proved that VNS and ta-VNS can inhibit the levels of serum proinflammatory cytokines, such as TNF-α, IL-1β, and IL-6, and the expression of NF-κB p65 in lung tissue. Caravaca et al. (26) found that 1-min VNS significantly reduced the total area of inflammatory lesions in the small intestine in a rat model of indomethacin-induced intestinal inflammation. Moreover, VNS significantly reduced the intestinal lesion area at 48 h when indomethacin administration was delayed. At the same time, they also found that the mechanism of VNS does not depend on the influence of the spleen. Another study (27) found that VNS can reduce plasma TNF-α, IL-1β, IL-6 and myeloperoxidase levels in TNBS-induced colitis rats through the autonomic nervous pathway. Clinical studies have also confirmed that VNS is expected to become an emerging method for the treatment of inflammatory bowel disease. Bonaz et al. (28) reported for the first time that after 7 patients with active Crohn's disease (CD) were treated with invasive VNS, the Crohn 's disease activity index (CDAI) was significantly improved in 5 patients, serum CRP and fecal calprotectin levels were significantly reduced, and vagus nerve tension was restored. Benjamin Sahn et al. found that taVNS significantly alleviates clinical symptoms and reduces fecal calprotectin levels in children and young adults with mild to moderate IBD during a concept validation clinical trial, thereby suggesting the anti-inflammatory effects of taVNS on this patient population (29). In a 12-month pilot study, Sinniger et al. reported that VNS enables patients with moderate Crohn's disease to restore steady-state vagal tone while concurrently reducing inflammatory markers, such as CRP and pro-inflammatory cytokines, including IL-6, IL-12, IL-23, and TNF-α (30).

### 3.5 Advantages and targets of taVNS

However, VNS, as a neuromodulation technique, can be categorized into invasive and non-invasive approaches. Invasive VNS typically involves the surgical implantation of electrodes and pulse generators directly onto the vagus nerve within the body, enabling automatic therapeutic stimulation through specific parameters and modes. Non-invasive VNS primarily refers to transcutaneous non-invasive vagus nerve stimulation (tVNS), which applies electrical stimulation non-invasively to the skin overlying the vagus nerve using surface electrodes. taVNS is one of neuromodulation techniques. Compared with invasive techniques, tVNS demonstrates advantages such as enhanced safety, reduced cost, and minimal invasiveness. Consequently, non-invasive VNS techniques like taVNS are more practical for the prevention and treatment of POFS in clinical settings due to their simplicity, cost-effectiveness, lack of invasiveness, and minimal potential side effects. In clinical applications, taVNS can be administered by placing a transauricular vagus nerve stimulator with a non-insulated film in the left ear's cymba conchae. The left ear is chosen because the efferent vagus nerve fibers innervating the heart are predominantly located on the right side. Yakunina et al. (31) investigated the effects of stimulating various vagus nerve distribution areas in the ear among healthy subjects. They compared four stimulation sites: the inner tragus, inferoposterior wall of the ear canal, cymba conchae, and earlobe (sham). Their findings revealed that stimulation of the cymba conchae produces the strongest and most extensive effects.

## 4 Evaluation of the hypotheses

Prospective studies can validate this hypothesis by utilizing the non-invasive nature of taVNS in anesthetized patients. The proposed scheme represents our hypothetical verification protocol, which has yet to commence. This study will include elderly patients undergoing colorectal cancer surgery, all of whom will be administered general anesthesia. The inclusion criteria for this study are outlined as follows: ASA I-II; aged 65–80 years; voluntary participation and signed informed consent. Patients with severe pulmonary hypertension, arrhythmia, or cardiac insufficiency, liver and kidney dysfunction, increased intracranial pressure, or intraocular pressure, recent use of analgesic or sedative drugs, previous chronic pain, mental disorders, or alcohol abuse history, allergies to the drugs used in the study, pregnancy, or lactation status, participation in other treatments within the past 6 months without consent will be excluded from the study. Additionally, patients who are deemed inappropriate by other clinical responsible doctors or competent doctors will also be excluded. After all patients have entered the room, the internal jugular vein and upper extremity venous access will be opened, and routine monitoring of electrocardiogram, invasive and non-invasive blood pressure, pulse oxygen saturation (SpO_2_), and nasal oxygen will be performed at 5 L/min. Anesthesia will be induced with dexamethasone 5 mg, lidocaine 40 mg, sufentanil 0.4 μg/kg, etomidate 0.3 mg/kg (or propofol 1–1.5 mg/kg), and cisatracurium 0.15 mg/kg (or rocuronium 0.9 mg/kg). After muscle relaxation is achieved, tracheal intubation (male: 7.0 # 10 Fr; female: 6.5 # 10Fr) will be performed under a visual laryngoscope, with mechanical ventilation, an inspiratory-to-expiratory ratio of 1:2, a tidal volume of 6–8 ml/kg, a respiratory rate of 12–14 times/min, and end-tidal carbon dioxide partial pressure (PETCO_2_) will be maintained at 35–45 mmHg. For anesthesia maintenance: Propofol (4–12 mg/kg/h), remifentanil (0.02–0.2 μg/kg/min), vasoactive drugs (specific to a drug) will be intermittently injected according to blood pressure and heart rate parameters to maintain blood pressure and heart rate within ±20% of the baseline value.

The patients will be randomly allocated into two groups: the taVNS group and the sham taVNS group, following a prospective, randomized, double-blind study design. After anesthesia induction in all patients, the stimulation electrode will be placed in the left cymba conchae and connected to the stimulation generator. Prior to the commencement of surgery, patients in the taVNS group will receive vagal nerve stimulation at a frequency of 25 Hz and a bidirectional pulse width of 100 μs for a duration of 30 min. In contrast, patients in the sham taVNS group will undergo identical stimulation parameters as the taVNS group for only 30 s before the device is turned off. The sham device will remain attached for 30 min to maintain blinding. Following the operation, the stimulation electrode will be removed from all patients. To ensure the integrity of the double-blind procedure during data collection, observers will remain outside the operating room whenever the stimulation generator is visible, and the external visual field will be obscured using a paper curtain.

Regarding the selection of VNS parameters in the program: Ashraf N. H., Gerges et al. (32) conducted a comprehensive review summarizing 109 studies on the clinical application of taVNS and identified inconsistencies in the parameters utilized, which complicates the determination of optimal settings. Consequently, the stimulus parameters selected herein are based on commonly adopted values in relevant research and are subject to adjustment according to the latest advancements in future studies.

The main observation indicators will include: ICFS-10 score, TNF-α levels, and IL-6 concentrations. These parameters will be assessed at the following time points: 1 day prior to the operation, at the conclusion of the operation, and on postoperative days 1, 3, and 7. The ICFS-10 is a self-administered questionnaire consisting of 10 items. Items 1 through 7 are scored using a six-point Likert scale (with items 1, 3, and 7 being reverse-scored), while items 8 through 10 utilize a five-point Likert scale. An ICFS-10 score exceeding 24 points will indicate the presence of POFS, with higher scores reflecting greater fatigue severity. The incidence of POFS will be evaluated 1 day before the operation and on postoperative days 1, 3, and 7, with the incidence of POFS at postoperative days 1, 3, and 7 being specifically quantified.

Reasons for the selection of observation indicators: Nøstdahl et al. (33) extracted 10 items from the International Classification of Functioning, Disability and Health Survey (ICFS) to construct a concise perioperative fatigue assessment scale (ICFS-10). The cut-off value for POFS in postoperative patients was determined to be 24 points, which could serve as an early warning indicator for potential adverse physiological and psychological effects associated with POFS. This scale demonstrates excellent reliability and validity in effectively evaluating POFS. TNF-α and IL-6 are two highly sensitive inflammatory mediators that play critical roles in reflecting the severity of the inflammatory response, particularly during its early stages following surgical stress (34). Wu et al. (35) demonstrated a significant correlation between IL-6 levels and postoperative fatigue. Furthermore, a meta-analysis comparing open and laparoscopic colorectal cancer surgeries revealed a strong association between serum TNF-α levels and early postoperative fatigue (5).

## 5 Discussion

Currently, the underlying mechanism of postoperative fatigue remains incompletely understood (36). Following surgery, individuals often employ psychological, pharmacological, exercise, and other interventions to alleviate postoperative fatigue (37). Ongoing research has shed light on the involvement of various inflammatory mediators in the pathophysiology of postoperative fatigue. Strategies aimed at blocking or reducing perioperative inflammatory responses may represent a promising approach for preventing and treating this condition. Although substantial research evidence indicates that certain anesthetic drugs, such as esketamine, ketamine, dexmedetomidine, and traditional Chinese herbal medicine, may effectively reduce the incidence of POFS (38–41), limited studies have systematically investigated the role of VNS in this context. In addition to activating afferent vagus nerve signals that impact brain function, therapeutic VNS exerts inhibitory effects on pro-inflammatory cytokine production (17). These findings suggest that controlling inflammation through CAP stimulation of the vagus nerve could be an effective method for improving postoperative fatigue. Given its direct and rapid endogenous mechanism, CAP can effectively suppress uncontrolled inflammation with a faster response compared to humoral anti-inflammatory pathways (14).

The high prevalence of POFS in elderly patients following colorectal cancer surgery led us to focus on this demographic for our investigation. The equilibrium between pro-inflammatory and anti-inflammatory agents is pivotal in postoperative recovery. Correcting the imbalance of these cytokines associated with significant surgical trauma and restoring immune homeostasis can effectively prevent and manage POFS. Drawing on prior research on CAP interventions in various pathological conditions, we propose that incorporating taVNS during surgery may reduce POFS by modulating the balance of pro-inflammatory and anti-inflammatory cytokines. In conclusion, the discovery of the CAP opens avenues for innovative therapies targeting inflammatory responses, with taVNS showing promise as a valuable clinical strategy for alleviating POFS in elderly patients post-colorectal cancer surgery.

## Data Availability

The original contributions presented in the study are included in the article/supplementary material, further inquiries can be directed to the corresponding authors.
